# Financial Inclusion and Its Impact on Health: Empirical Evidence From Asia

**DOI:** 10.3389/fpubh.2022.948964

**Published:** 2022-07-01

**Authors:** Wenling Xiao, Ran Tao

**Affiliations:** ^1^School of Economics, Shandong Women's University, Jinan, China; ^2^Qingdao Municipal Center for Disease Control and Prevention, Qingdao, China

**Keywords:** human health, financial inclusion, FDI, GDP, Asian

## Abstract

Asian countries have shown remarkable progress in financial inclusion and have become the world's fastest-growing regions. However, the financial inclusion-human health nexus has not received much attention. This study contributes to the empirical literature by examining the effect of financial inclusion on population health using panel data from Asian countries from 2007 to 2019. Population health is measured by death rate and life expectancy at birth. Our study finding shows that digital financial inclusion increases life expectancy but decreases the death rate in Asia. At the same time, financial inclusion positively impacts life expectancy and has a negative impact on the death rate in Asia. Finding also suggests that Internet users, GDP, and FDI have improved population health by increasing life expectancy and decreasing the death rate. The results suggest some essential policy implications.

## Introduction

Financial inclusion provides affordable, accessible, and beneficial products and financial services to individuals and businesses responsibly and sustainably (1, 2). Financial development denotes improvement in the size, stability, and efficiency of the financial system. While financial inclusion denotes those individuals and businesses are able to fulfill their requirements due to accessibility to affordable financial services and products (3). Recently, efforts to promote financial inclusion have enlarged. Financial inclusion is considered the fundamental tool that can be used to obtain social and economic development, especially in vulnerable societies (4). World Bank (1) declared financial inclusion as enabling most Sustainable Development Goals (SDGs). Access to finance simplifies daily activities and planning for long-term goals and emergencies for families and businesses.

United Nations Capital Development Fund (UNCDF) implies that financial account holders can access credit easily, enlarge and retain their businesses, invest in education and health, and handle financial shocks that enhance livelihoods' sustainability (5). Financial inclusion directly influences the health of people (6). Due to the occurrence of highly unpredictable diseases, financial inclusion can support individuals in bearing these treatment expenses through savings that lead to better health outcomes (7, 8). Furthermore, financial inclusion helps people afford better quality health inputs, such as a nutritious diet, clean energy, and improved sanitation (9). Besides these, financial inclusion reduces mental stress by providing financial stability that could end up in good quality health outcomes (10, 11). Various studies have explored the impact of financial inclusion on social and economic indicators (12–14), and very few studies have explored the impact of financial inclusion on human health (15, 16). Literature discloses that high mortality is considered a measure representing the bad quality of human health, and enlarged life expectancy is a measure of good quality of human health.

As far as the theoretical aspect of financial inclusion is concerned, literature provides two theories regarding financial inclusion: the vulnerable group theory and the public goods theory of financial inclusion (17). The vulnerable group theory implies that financial inclusion should consider a vulnerable population of society, including the poor, younger, older, and women (6). In contrast, the public goods theory claims that financial inclusion should be accessible to the whole society and no one should be left excluded (18). However, the theory of capability implies that financial inclusion enlarges the freedom of people in making choices for essential necessities such as good quality healthcare, education, clean water, and sanitation facilities that improve the health outcomes of people (19).

As long as the empirical aspect of the nexus between financial inclusion and health is concerned, Claessens and Feijen (20) found that credit to the private sector is positively linked with human health. In the case of South Africa, Sarma & Pais (21) found a strong association between financial development and life expectancy. Their study measures financial development by domestic credit as a percent of GDP, M3 as a percent of GDP, and domestic credit to the private sector as a percent of GDP (22). In the case of OECD economies, Gunakar (23) found that financial development enhances health outcomes by increasing the extent of life expectancy and reducing the rate of infant mortality. Financial development in this study is measured by liquid liabilities as a percent of GDP, credit to the private sector as a percent of GDP, and market capitalization as a percent of GDP (24). In the case of African economies, Chireshe (25) found that financial development increases life expectancy and reduces the child mortality rate. Gyasi et al. (15) explored the impact of financial inclusion on adult health in the case of Ghana. It is reported that financial inclusion is positively related to the health outcomes of adults (26). However, despite much effort, we cannot find any study exploring the impact of financial inclusion on human health in the case of Asian economies (27). This study provides us answer to the following question: Does financial inclusion lead to better health outcomes? To our knowledge, this is the first study of its kind that determines the nexus between financial inclusion and health outcomes (18).

Given this lacuna of existing literature, our study investigates the impact of financial inclusion on public health in the case of selected Asian economies. The sample of the study is selected based on data availability (28). Our study will make contributions to the existing literature in the following manners. Firstly, to the best of the authors' knowledge, this study is the first one exploring the nexus between financial inclusion and public health in the case of the Asian region. Secondly, the study will use 2 SLS and GMM approaches to explore this nexus from 2007 to 2019 (29). Thirdly, this is the first-ever study in the Asian region covering proxy health measures such as life expectancy and death rate. Lastly, most previous studies measure financial development through domestic credit to the private sector as a percent of GDP (26). However, our study measures financial inclusion using two proxy measures, namely ATMs and debit cards. This study tries to deal with the endogeneity issues and perform sensitivity analysis to check the robustness of the outcomes. The findings of the study will support policymakers in designing such policies that ease the involvement of individuals in financial activities to protect their health outcomes.

## Model and Methods

In recent years, financial inclusion has been supposed as a dynamic tool for attaining human development in advanced and developing countries (30). Financial inclusion also improves macroeconomic stability and inclusive economic growth (2). Our study is based on the vulnerable group theory (17). Theoretical developments have argued that financial inclusion improves human development. As such, we employ the following economic model that follows (31):


(1) $$\begin{array}{l} Health_{it}=\;\eta_{0}+\;\eta_{1}FI_{it}+\eta_{2}Internet_{it}+\eta_{3}HE_{it}\\ \;+\eta_{4}GDP_{it}+\eta_{5}FDI_{it}+\lambda_{i}+\varepsilon_{it} \end{array}$$

where is the population health (*Health*_*it*_) that depend on financial inclusion (*FI*), internet users (Internet), health expenditure (HE), GDP growth (GDP), and foreign direct investment (FDI). Where *λ*_*i*_ refers to unobserved individual-country and *ε*_*it*_ is the error term. However, *i*(t) represents the country (year), and the remaining *η*s are coefficients of the concerned explanatory variables. Financial inclusion can significantly improve human health outcomes. Thus, we expect an estimate of d to be positive. Following the research work of Immurana et al. (32), the control variables included in the health model include internet users, health expenditure, GDP growth, and FDI. The remaining explanatory variables have a favorable impact on population health; thus, estimates of *η*_2_, *η*_3_, *η*_4_, and *η*_5_ are expected to be positive. We estimate model (1) using the two-stage least squares (2 SLS) technique. This method is best suited because it can easily address the problem of endogeneity. The main sources of endogeneity are measurement errors, omitted variable bias, and reverse causality. These issues arise for different reasons; however, they can overcome the problem using instrumental variables. For estimation, this study employs the 2 SLS estimators to estimate the baseline outcomes. In our model, financial inclusion is a potential endogenous variable. The augmented panel model is:


(2) $$\begin{array}{l} Health_{it}=\;\eta_{0}+\;\eta_{1}Health_{it-1}+\;\eta_{1}FI_{it}+\eta_{2}Internet_{it}\\ \;+\eta_{3}HE_{it}+\eta_{4}GDP_{it}+\eta_{5}FDI_{it}+\lambda_{i}+\varepsilon_{it} \end{array}$$

while *Health*_*it*−1_ is the first lag of health outcomes in equation (2), which is a dynamic term in the panel model. We estimate model (2) using the Blundell & Bond (33) system GMM technique. The system GMM approach has been used in many previous empirical health-related studies (32). Following Immurana et al. (31, 34), we use the dynamic panel-data model (2). This econometric specification is widely used in the empirical finance literature to examine the nexus between financial inclusion and human development. This approach is suitable as the number of countries (*N* = 18) is more than the number of years (*T* = 13), as in our study. Few diagnostics tests, such as the serial correlation test and the Sargan test statistic—are also used to demonstrate the validity of estimates.

## Data

Table 1 displays the details of descriptive statistics of variables, definitions and symbols of variables, and sources of data series. The list of selected Asian countries is reported in Table 2. Asian Health in this study is measured by two indicators such as life expectancy and death rate. Two indicators also measure financial inclusion: ATMs per 1,000 adults and debit cards (% age 15+). Previous studies have used the same variables for financial inclusion (2, 35). The role of internet use, health expenditures, foreign direct investment, and GDP growth have been added as control variables. Internet use is measured as internet users in the percentage of the population. Health expenditures are measured as a percentage of GDP. GDP growth is taken in annual percentage. Net inflows determine FDI as a percent of GDP. The data for financial inclusion indicators have been taken from IMF, while the data for the remaining variables have been collected from the World Bank. Table 2 shows that the mean (standard deviation) for life expectancy is 72.4 (44.7), the death rate is 6.57 (1.95), ATMs is 53.0 (39.4), a debit card is 30.7 (24.0), the internet user is 36.7 (27.4), health expenditure is 4.33 (1.38), GDP growth is 5.38 (2.85), and FDI is 4.46 (3.99). While Table 3 shows that the correlation matrix and findings is free from multicollinearity problem.

**Table 1:** Descriptive statistics and definitions.

**Variable**	**Mean**	**Std. dev**.	**Definitions**	**Sources**
LE	72.4	44.7	Life expectancy at birth, total (years)	World bank
DR	6.57	1.95	Death rate, crude (per 1,000 people)	World bank
ATMs	53.0	39.4	ATMs per 100,000 adults	IMF
Debit	30.7	24.0	Debit card (% age 15+)	IMF
Internet	36.7	27.4	Individuals using the Internet (% of the population)	World bank
HE	4.33	1.38	Current health expenditure (% of GDP)	World bank
GDP	5.38	2.85	GDP growth (annual %)	World bank
FDI	4.46	3.99	Foreign direct investment, net inflows (% of GDP)	World bank

**Table 2:** List of countries.

**1**	**Bangladesh**	**7**	**Indonesia**	**13**	**Korea, Rep**.
**2**	**India**	**8**	**Malaysia**	**14**	**Russian Federation**
**3**	**Pakistan**	**9**	**Philippines**	**15**	**Kazakhstan**
**4**	**Sri Lanka**	**10**	**Singapore**	**16**	**Kyrgyz Republic**
**5**	**China**	**11**	**Thailand**	**17**	**Tajikistan**
**6**	**Mongolia**	**12**	**Vietnam**	**18**	**Uzbekistan**

**Table 3:** Matrix of correlations.

	**LE**	**DR**	**ATMs**	**Debit**	**Internet**	**HE**	**GDP**	**FDI**
LE	1							
DR	−0.334	1.000						
ATMs	0.554	0.203	1.000					
Debit	0.596	0.063	0.641	1.000				
Internet	0.697	0.031	0.693	0.683	1.000			
HE	0.209	0.052	0.296	0.115	0.254	1.000		
GDP	−0.163	0.253	−0.370	−0.194	−0.282	−0.068	1.000	
FDI	0.194	0.118	−0.080	0.268	0.153	0.006	0.222	1.000

## Results and Discussion

Table 4 reports the results of 2 SLS and GMM estimates for life expectancy models. It is found that ATMs and life expectancy are significantly and positively associated in both 2 SLS and GMM models. It reveals that a 1 percent upsurge in the number of ATMs improves life expectancy by 0.134 percent in the 2 SLS model and 0.051 percent in the GMM model. The findings further reveal that debit card and life expectancy are also significantly and positively associated in both 2 SLS and GMM models. It implies that a 1 percent upsurge in the number of credit cards improves life expectancy by 0.091 percent in the 2 SLS model and 0.024 percent in the GMM model. Hence, it is confirmed that both financial inclusion indicators contribute significantly to enhancing population health in selected 18 Asian economies. This finding is supported by Immurana (32), who noted that financial inclusion enhances health in Africa. This finding infers that digital financial inclusion easy financial services, enabling people to acquire health-related goods and services. This means that financial services boost human health. This finding is also backed by Ofosu-Mensah Ababio et al. (34), who reported that financial inclusion is an effective tool for achieving socio-economic development by reducing poverty and income inequality. The findings validate the study of Churchill et al. (36) that shows that financial inclusion has a strong poverty-reducing effect, improving population health. Another possible reason is that financial inclusion improves human health via income channels. Financial inclusion prompts the human development process in Asian economies. Findings infer that a well-performing digital financial system is an important factor in human development.

**Table 4:** Financial inclusion and life expectancy (2 SLS & GMM).

	**2 SLS**		**2 SLS**		**GMM**		**GMM**	
	**Coef**.	**z-stat**	**Coef**.	**z-stat**	**Coef**.	**z-stat**	**Coef**.	**z-stat**
L.LE					0.901^**^	2.060	0.970^***^	4.930
ATMs	0.134^***^	4.070			0.051^****^	5.330		
Debit			0.091^***^	8.150			0.024^**^	2.210
Internet	0.059^*^	1.920	0.020^***^	2.950	0.021^**^	2.160	0.030^***^	4.650
HE	0.008	0.020	0.302	1.560	0.017^**^	2.030	0.010	1.180
GDP	0.059	1.020	0.029	1.030	0.012^***^	4.580	0.013^***^	4.800
FDI	0.045^*^	1.820	0.036^***^	2.740	0.015^***^	4.280	0.013^***^	2.970
Cons	6.970^***^	6.660	7.226	9.210	3.089^***^	6.720	2.522^***^	8.290
Observations	234		234		216		216	
Countries	18		18		18		18	
AR (1)					0.301		0.102	
AR (2)					0.210		0.087	
Sargan-test					0.254		0.345	

*** *Significant at 1%;*

** *Significant at 5%;*

* *Significant at 10%*.

The impact of internet use on life expectancy is found to be significant and positive on life expectancy in all four models, displaying that the use of the internet tends to improve human health in the sample of selected Asian economies. This result is in line with Majeed & Khan (37), who found that internet development improves population health by increasing financial and health literacy, spreading health information, and health care services. This finding is also supported by Mushtaq & Bruneau (38), who noted that the composite impact of internet development and financial inclusion is an important factor for human development. The findings display that the nexus between health expenditures and life expectancy is significantly positive only in one model, confirming that current health expenditures are capable to improve health outcomes in Asian economies. The GDP and life expectancy association is found significantly positive in both GMM models, showing that an upsurge in GDP improves public health in Asian economies. Ordinarily, economic progress is found to improve human health by increasing positive externalities. For instance, Woodward et al. (39) found economic development to boost human health. The impact of FDI on life expectancy is found to be significantly positive in all four models confirming that FDI plays a prominent role in improving people's health in Asian economies. Thus, it is confirmed that financial inclusion, internet use, GDP, and FDI are significant indicators of human health in the case of Asian economies. Both GMM models are correctly specified, as confirmed by a statistically insignificant coefficient estimate of the Sargan test.

Table 5 reports the results of 2 SLS and GMM estimates for death rate models. It is reported that ATMs and death rates are significantly and negatively associated in both 2 SLS and GMM models. It implies that a 1 percent upsurge in ATMs users reduces the death rate by 0.008 percent in the 2 SLS model and 0.007 percent in the GMM model. The findings display that debit card and death rate are associated significantly and negatively in the GMM model only, while the association is found statistically insignificant in the case of the 2 SLS model. It displays that a 1 percent rise in the number of credit cards reduces the death rate by 0.002 percent in the GMM model. Thus, the findings of both 2 SLS and GMM models confirmed that both determinants of financial inclusion, ATMs and credit card, play a significant role in improving population health in Asian economies. The nexus between internet use and the death rate is found significant and negative in the case of two models, revealing that internet use plays a prominent role in improving human health in selected Asian economies. The nexus between health expenditures and the death rate is found significant and negative in all four models, displaying that current health expenditures play a fundamental role in improving population health in Asian economies. The nexus between GDP and death rate is significantly negative in the case of both GMM models, displaying that increase in GDP significantly improves public health in selected Asian economies. The association between FDI and death rate is significantly negative in the case of three models, inferring that FDI plays a key role in the improvement of population health in the case of Asian economies. Similar to the life expectancy model, financial inclusion, internet use, GDP, and FDI are significant determinants of human health in the sample of selected 18 Asian economies. The statistically insignificant coefficient estimate of the Sargan test confirms that both GMM models are correctly specified.

**Table 5:** Financial inclusion and death rate (2 SLS & GMM).

	**2 SLS**		**2 SLS**		**GMM**		**GMM**	
	**Coef**.	**z-stat**	**Coef**.	**z-stat**	**Coef**.	**z-stat**	**Coef**.	**z-stat**
L.DR					0.952^***^	9.292	0.953^***^	8.122
ATMs	−0.008^**^	2.200			−0.007^***^	3.160		
Debit			−0.005	1.130			−0.002^***^	3.460
Internet	−0.004	0.640	−0.009^***^	3.020	−0.005	0.320	−0.007^**^	2.130
HE	−0.200^***^	2.850	−0.218^***^	2.630	−0.019^**^	2.370	−0.017^**^	2.050
GDP	−0.012	0.546	−0.014	0.150	−0.015^**^	1.980	−0.016^***^	2.450
FDI	−0.008	1.430	−0.013^**^	2.370	−0.005^**^	2.340	−0.004^***^	3.370
Cons	−6.252^***^	21.62	−6.058^***^	17.91	−6.872	1.590	−0.793	0.180
Observations	234		234		216		216	
Countries	18		18		18		18	
AR (1)					0.422		0.321	
AR (2)					0.210		0.321	
Sargan-test					0.345		0.412	

*** *Significant at 1%;*

** *Significant at 5%*.

## Conclusion and Implications

In this study, an effort is made to explore the nexus between financial inclusion and population health in the case of selected Asian economies over the time span of 1995–2020. Financial inclusion is measured through ATMs and debit cards in this study, while health is measured through death rate and life expectancy. For estimation purposes, the 2 SLS and GMM methods have been used. The obtained results are as follows. Both ATMs and credit cards positively affect population health, revealing that financial inclusion enhances population health in Asian economies. Other control variables such as GDP, current health expenditures, FDI, and internet use positively influence human health as described in most cases.

Thus, the study put forward some important policy implications for policymakers, stakeholders, and governments of Asian economies. It is suggested that the enlargement of financial inclusion should be the responsibility of governments, stakeholders, potential customers, service providers, financial supervisors, financial regulators, and development agencies. The promotion of financial inclusion should be embarked by the whole banking sector to further improves human health. New savings or deposit methods through branchless avenues and technological methods must be encouraged to support customers in accessing and depositing money. The governments should start initiatives that provide financial education and training to individuals about using branchless and digital avenues. Another suggestion is that there should be strong collaborations and linkages among financial service providers, financial regulations, and governments. The restrictions on inflows of FDI should be relaxed. Governments should establish strong regulatory and law enforcement organizations. Remote and backward areas should be modernized by establishing improved physical infrastructures such as telecommunication, electricity, and paved roads that provide mental peace to people, thus improving their health and livelihood. The stakeholders and governments should struggle to guarantee financial inclusion services to individuals from both supply and demand sides to enhance the health and wellbeing of people in Asian economies.

Besides these implications, the study also faces some limitations that must be considered in future studies. For instance, the study has used only two indicators to measure human health; however, there are several other health indicators that must be considered in future research, such as mental health, other chronic diseases, and maternal health. Our study is limited to the Asian region and adopts a linear method of estimation to explore the nexus between financial inclusion and human health. However, future studies can adopt non-linear methods of estimation to get more interesting results. Furthermore, future studies can also replicate these analyses for other regions and economies.

## Data Availability Statement

Publicly available datasets were analyzed in this study. This data can be found here: https://data.worldbank.org/.

## Author Contributions

WX: conceptualization, software, data curation, and writing—original draft preparation. RT: methodology and writing—reviewing and editing. WX and RT: visualization and investigation. Both authors contributed to the article and approved the submitted version.

## Funding

This study was supported by High-level Talent Introduction Research Project of Shandong Women's University (Grant No. 2021RCYJ03) and Cultivation Fund for High-level Scientific Research Projects of Shandong Women's University (Grant No. 2021GSPSJ05).

## Conflict of Interest

The authors declare that the research was conducted in the absence of any commercial or financial relationships that could be construed as a potential conflict of interest.

## Publisher's Note

All claims expressed in this article are solely those of the authors and do not necessarily represent those of their affiliated organizations, or those of the publisher, the editors and the reviewers. Any product that may be evaluated in this article, or claim that may be made by its manufacturer, is not guaranteed or endorsed by the publisher.
